# Population genetics of *Camellia granthamiana*, an endangered plant species with extremely small populations in China

**DOI:** 10.3389/fgene.2023.1252148

**Published:** 2023-10-05

**Authors:** Sufang Chen, Wenyan Li, Wei Li, Zhongcheng Liu, Xianggang Shi, Yanli Zou, Wenbo Liao, Qiang Fan

**Affiliations:** ^1^ State Key Laboratory of Biocontrol and Guangdong Provincial Key Laboratory of Plant Resources, School of Life Sciences, Sun Yat-sen University, Guangzhou, China; ^2^ Shenzhen Dapeng Peninsula National Geopark, Shenzhen, China; ^3^ College of Forestry and Landscape Architecture, South China Agricultural University, Guangzhou, China; ^4^ School of Ecology, Sun Yat-sen University, Shenzhen, China; ^5^ Shenzhen Academy of Environmental Sciences, Shenzhen, China

**Keywords:** *Camellia granthamiana*, restriction-site-associated DNA sequencing, population genomics, extremely small populations, shallow-genome sequencing, Guangdong, Theaceae

## Abstract

**Introduction:** *Camellia*, the largest genus of Theaceae, is well-known for having high economic values. *Camellia granthamiana* demonstrates large beautiful flowers with some primitive characters, such as multiple large and persistent bracteoles and sepals, was listed as Vulnerable species on the IUCN Red List.

**Methods:** In this study, we investigated all possible records of the species, and sampled four natural populations and five cultivated individuals. By applying shallow-genome sequencing for nine individuals and RAD-seq sequencing for all the sampled 77 individuals, we investigated population genetic diversity and population structure of the species.

**Results and discussion:** The results showed that the population sampled from Fengkai, previously identified as *C. albogigias*, possessed different plastid genome from other species possibly due to plastid capture; the species possesses strong population structure possibly due to the effect of isolation by distance, habitat fragmentation, and self-crossing tendency of the species, whose effective population size declined quickly in the past 4,000 years. Nevertheless, *C. granthamiana* maintains a medium level of genetic diversity within population, and significant differentiation was observed among the four investigated populations, it is anticipated that more populations are expected to be found and all these extant populations should be taken into instant protection.

## 1 Introduction

*Camellia*, the largest genus of Theaceae, contains approximately 280 species distributed near the Tropic of Cancer in East Asia, and 238 species were recorded in China, which are mainly distributed in Yunnan, Guangxi, Guangdong, and Sichuan provinces (Chang, 1998). *Camellia* is well known for its high economic values (such as *C. sinensis* (Linnaeus) Kuntze, known as China tea, and *C. oleifera* C. Abel, as an important source of oil) and ornamental values (such as *C. japonica* Linnaeus, widely cultivated for beautiful flowers).

Karyotypic studies on *Camellia* (Chang, 1998; Li, 2001) showed that the number of chromosomes of most *Camellia* species was estimated to be 30, with some exceptions such as *Camellia albogigas* Hu and *Camellia granthamiana* Sealy (2n = 4x = 60) and *C. vietnamensis* and *C. grandiflora* (2n = 8x = 120). Both *C*. *granthamiana* and *C. albogigas* were ascribed to the sect. *Archecamellia* Sealy of subgen. *Protocamellia* Chang in *Flora Reipublicae Popularis Sinicae*, as they demonstrate some primitive characters, such as multiple large and persistent bracteoles and sepals (Chang, 1998). Geographically, *C*. *albogigas* was recorded only in Fengkai, Guangdong, while *C. granthamiana* was recorded in the western and eastern areas of Guangdong and Hong Kong, China. Currently, *C. albogigas* is treated as a synonym of *C. granthamiana* in the *Flora of China* (Min and Bartholomew, 2007) and *C. granthamiana* is listed as vulnerable on the IUCN Red List (http://www.iucnredlist.org).

For the better understanding of threats to endangered species, phylogenetics and population genetics have been widely used in conservation genetics and (or) conservation biology to investigate the causes of species decline, such as habitat fragmentation, genetic drift, and barriers to gene flow, and identify counteracting measures and conservation units. However, recent advancements in genomic tools have further enabled the utilization of vastly expanded datasets of single-nucleotide polymorphisms (SNPs), providing unprecedented insights into the importance of genetic diversity in conservation (Willi, et al., 2022). Currently, we are reviewing all specimen records in the Chinese Virtual Herbarium (https://www.cvh.ac.cn/) and the online Flora of China (http://www.iplant.cn/). We have successfully collected three populations of *C. granthamiana* and one population of *C. albogigas* in Guangdong, China. Furthermore, we utilized the shallow-genome sequencing method to *de novo* assemble the chloroplast genomes and construct a plastid phylogenetic tree. At the same time, we performed the restriction-site-associated DNA sequencing method on all the sampled populations. Through these efforts, we endeavored to investigate whether *C. albogigas* and *C. granthamiana* constitute one species or two, assess the population structure and genetic diversity of these species, and eventually provide some valuable information for their conservation.

## 2 Materials and methods

### 2.1 Sample collection and Illumina sequencing

**FIGURE 1:**
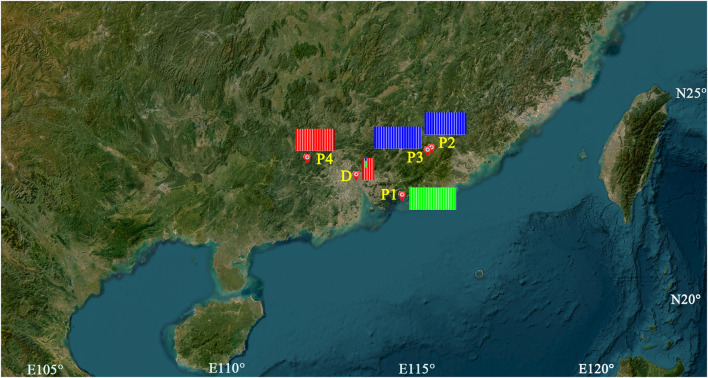
Geographical locations and ADMIXTURE analysis of the samples of *C. granthamiana* and *C. albogigas*. P1, P2, P3, P4, and D: population ID. Map data: Google, TerraMetrics.

Fresh leaves were sampled for the three populations of *C. granthamiana* and one population of *C. albogigas.* For each population, geographical information was recorded using a Garmin GPS unit (GPSMAP 62sc, Shanghai, Table 1; Figure 1). Specimens were deposited in the herbarium of Sun Yat-sen (SYS) University, China. The type specimen of *C. granthamiana* was recorded in Hong Kong, China. In Sun Yat-sen University, five individuals of *C. granthamiana* or *C. albogigas* were cultivated: one was transplanted from Hong Kong, China (*C. granthamiana*), and the others were transplanted from Fengkai, Guangdong, China (*C. albogigas*). As it is difficult to make clear which individual was transplanted from Hong Kong, China, we sampled fresh leaves from all five individuals (Table 1).

**TABLE 1:** Geographical information for sampled populations of *C*. *granthamiana* and *C. albogigas.*

Population ID	Location	Latitude and longitude	*N*	*H*_*O*_	*H*_*E*_	*F*_*IS*_	Ind. ID
*Camellia granthamiana*							
P1	Qiniangshan	N22°31ʹ27.65ʺ, E114°32ʹ30.56ʺ	20	0.110	0.151	0.135	P1-1
P2	Zijin	N23°42ʹ29.13ʺ, E115°11ʹ54.25ʺ	20	0.102	0.147	0.143	P2-1
P3	Zijin	N23°44ʹ17.05ʺ, E115°18ʹ41.44ʺ	18	0.108	0.162	0.174	P3-1
*Camellia albogigas*							
P4	Heishiding	N23°26ʹ36.80ʺ, E111°56ʹ54.20ʺ	20	0.105	0.120	0.065	P4-1
Cultivar							
D	Sun Yat-sen University	N23°05ʹ44.75ʺ, E113°17ʹ30.54ʺ	5				D-1 … D-5

*N*, number of individuals; *H*_*O*_, observed heterozygosity; *H*_*E*_, expected heterozygosity; *F*_*IS*_, inbreeding coefficient; Ind. ID, ID of individuals for shallow-genome sequencing.

For each population, one individual was randomly selected for shallow-genome sequencing, while RAD-seq was performed on the four populations. For the five individuals sampled from the Bamboo Garden of Sun Yat-sen University, both RAD-seq and shallow-genome sequencing were performed. The fresh leaves were dried and preserved on silica gel in sealed bags and then sent to JieRui BioScience Co., Ltd. (Guangzhou, China) for DNA extraction, library preparation, and Illumina sequencing.

Briefly, genomic DNA for each sample was extracted using the modified CTAB method (Doyle and Doyle, 1987) and purified using magnetic beads. For shallow-genome sequencing, a library was constructed for each sample using the TruePrep DNA Library Prep Kit and then sent for Illumina sequencing on the NovaSeq 6000 platform according to the standard operation procedure. For RAD-seq, the purified genomic DNA was digested with EcoR I and Mse I restriction enzymes, barcodes and Illumina adapter sequences were ligated to the digested DNA fragments, and subset fragments with different barcodes were pooled together. Then, the pooled fragments were purified and size-selected to 350–550 bp in agarose, and these selected fragments were amplified using PCR. Finally, we removed residual primers and purified PCR libraries using magnetic beads, and Illumina sequencing was conducted on the NovaSeq 6000 platform according to the standard operation procedure.

### 2.2 Plastid genome assembly and phylogenetic tree construction

The raw data were produced from Illumina sequencing, and nine plastid genomes were successfully assembled using NOVOPlasty 2.7.2.pl (Dierckxsens, et al., 2017), in which the plastid genome *C. granthamiana* (NC_038181.1) was used as a reference and its *rbcL* gene was used as a seed. Furthermore, plastid genomes for 111 samples of *Camellia* and one sample of *Polyspora hainanensis* were downloaded from the NCBI website (https://www.ncbi.nlm.nih.gov/). In total, 120 plastid genome sequences were aligned using MAFFT, which performs well in reducing CPU time and increasing the accuracy of alignments for sequences with large insertions or extensions (Katoh et al., 2002). Sites with missing/ambiguous data and gaps were excluded using MEGA X (Kumar et al., 2018), and a phylogenetic tree based on the maximum likelihood method was constructed using IQ-TREE v2.2.0 (Nguyen, et al., 2015) by setting “-m MFP -bb 2000.”

### 2.3 RAD-seq data processing and population analyses

The raw reads produced from the Illumina platform were processed using Stacks 2.55 software (Catchen, et al., 2013). Initially, the procedure *process_radtags* was used to demultiplex RAD tags, and five samples were randomly selected from each population to determine the optimal values for M (number of mismatches allowed between stacks within samples) and n (number of mismatches allowed between stacks between samples). Then, the Perl script *denovo-map*.pl was applied to process all the samples, and the procedure *populations* was used to filter the results by setting “--min-maf 0.05 --max-obs-het 0.8 -R 0.6 –write-random-snp,” in which one random SNP was extracted for each locus.

Principal coordinate analysis (PCA) was performed using PLINK v1.90 (Chang, et al., 2015), and a Python script was used to draw the scatter diagram. Then, Bayesian cluster analysis was conducted using ADMIXTURE v1.3.0 software (Alexander, et al., 2009). The number of groups (*K*) was set in the range of 2–6, and the optimal *K* was determined with the lowest cross-validation (CV) error. The produced VCF file was transformed to a PHY file using the Python script vcf2phylip.py (Ortiz, 2019), and a phylogenetic tree was constructed using the IQ-TREE program by setting “-m MFP -bb 2000.”

Applying the software Arlequin (Excoffier and Lischer, 2010), an analysis of molecular variance (AMOVA) was performed. In this analysis, four populations were assigned to three groups according to PCA and ADMIXTURE analysis, pairwise *F*_*ST*_ was calculated based on the Kimura 2P method, and *N*_*M*_ was estimated based on the formula *N*_*M*_ = 1/(4*F_*ST*_ + 1). The Mantel test was performed using GenAlEx 6.5 (Peakall and Smouse, 2012) to calculate the correlation between genetic distance *F*_*ST*_/(1-*F*_*ST*_) and geographic distance (ln) by setting 999 permutations (Diniz, et al., 2013).

### 2.4 Historical demographic changes

Unfolded site frequency spectra (SFS) were generated using easySFS (https://github.com/isaacovercast/easySFS). The values were projected downward to maximize the number of segregating sites for both species. Stairway Plot v. 2.1.1 was used to infer recent historical dynamics of the effective population size of *C. granthamiana* and *C. albogigas* based on unfolded SFS (Liu and Fu, 2015). Using whole-genome sequencing, the nucleotide substitution rate of walnut was estimated to be 2.29 × 10^−9^ per site per year (Luo, et al., 2015), which is quite close to other woody perennials such as palms (2.61 × 10^−9^, Gaut et al., 1996), poplar (2.5 × 10^−9^, Buschiazzo, et al., 2012), 6.5 times slower than *Arabidopsis* lineage (1.5 × 10^−8^, Koch, et al., 2000), and 4.7 times slower than *Medicago* lineage (1.08 × 10^−8^, Young et al., 2011). These data suggested that the molecular clock in plants is related to the life-cycle length (Luo, et al., 2015). In this analysis, the mutation rate was set to 2.29 × 10^−9^ per site per year according to walnut, which starts to bear fruit in 4 years (Chen, 2020), and the generation time for *C. granthamiana* was set to 5 years based on field observations.

## 3 Results

### 3.1 Plastid phylogenetic tree

For shallow-genome sequencing, approximately 6 G raw data were produced for each sample, and nine plastid genomes were successfully assembled and circularized in this study (Table 1). The length of these plastid genomes was smallest for the three samples P2-1, P3-1, and D-2 (156,971–156,980 bp), at a medium level for the other five samples D-1, D-3, D-4, D-5, and P4-1 (157,001–157,010 bp), and was largest for the sample P1-1 (158,031 bp). Manual inspection of these nine plastid genome sequences showed no ambiguity at base “N,” and two heterozygous positions with base “M” were corrected to base “C” according to other plastid sequences.

**FIGURE 2:**
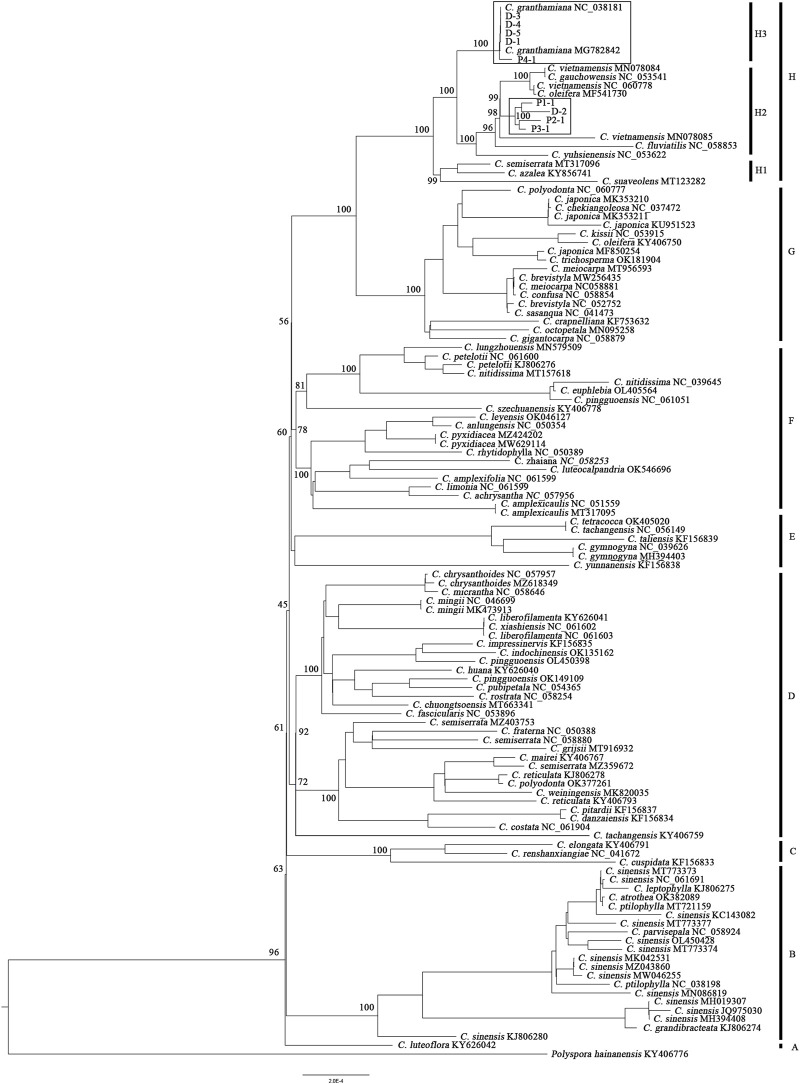
Phylogenetic tree of 72 *Camellia* species constructed from 120 plastid genomes based on the maximum likelihood method. At the nodes of the tree, maximum likelihood ultrafast bootstrap support values are shown for the main clades.

Together with the 111 plastid genomes downloaded from NCBI, the phylogenetic tree containing 72 *Camellia* species (Figure 2) could be further divided into eight clades (A–H), although relationships among these clades were not fully resolved with high support values. The representative sample of the population P4 (P4-1) and the four samples collected from Sun Yat-sen University (D-1, D-3, D-4, and D-5) clustered together with the two plastid genomes downloaded from NCBI with high support values (PP: 100), forming subclade H3. The three representative samples of the population P1–P3 (P1-1, P2-1, and P3-1) and one sample collected from Sun Yat-sen University (D-2) clustered together with the other five *Camellia* species, forming subclade H2; subclade H2 formed a sister relationship with H3 and then clustered together with subclade H1.

### 3.2 RAD-seq data processing

A total of 12 G raw data were produced for the 77 samples of *Camellia*, and the number of tags retained for each sample ranged from 178,956 to 3,101,602 (average number of tags: 2,240,608). After filtering with the program “*populations*,” a total of 9,036 loci containing 7,152 variant sites were retained for further analysis. The observed heterozygosity (*H*_*O*_) ranged from 0.102 to 0.110, the expected heterozygosity (*H*_*E*_) ranged from 0.120 to 0.162, and the inbreeding coefficient (*F*_*IS*_) ranged from 0.065 to 0.174 (Table 1).

### 3.3 Population structure

**FIGURE 3:**
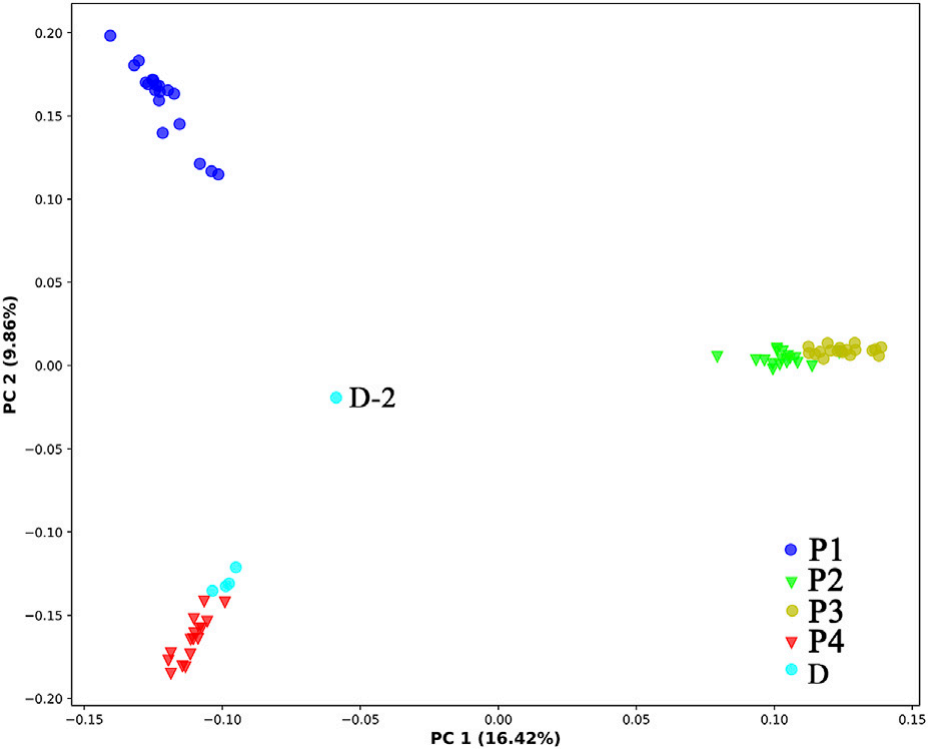
Principal coordinate analysis of the 78 individuals of *C*. *granthamiana* and *C. albogigas*. P1, P2, P3, P4, and D: population ID.

In PCA (Figure 3), the first, second, and third axes account for 16.42%, 9.86%, and 7.47% of total variances, respectively. The first and second axes divided the four natural populations into three clusters: P1 and P4 were ascribed to an independent cluster, respectively, while P2 and P3 were ascribed to the same cluster. Among the five individuals cultivated at Sun Yat-sen University, four shared the same cluster with P1, while the remaining one (D-2) remained separate from all other populations.

ADMIXTURE analysis (Figure 1) showed that the least value of CV was obtained when *K* = 3, P1 and P4 were assigned to one group each, and P2 and P3 shared the same group. Among the five individuals cultivated at Sun Yat-sen University, D-1, D-3, D-4, and D-5 were assigned to the same group as population P4 and the last individual was assigned to the admixture of the three groups. AMOVA showed that the genetic variation of *C. granthamiana* is primarily maintained within populations (44.02%, *F*_*ST*_ = 0.215, *p* < 0.001, Table 2).

**TABLE 2:** Analysis of molecular variance for the four populations of *C*. *granthamiana*.

Source of variation	d.f.	Sum of squares	Variance components	Percentage of variation	
Among groups	2	4,689.442	44.771	21.47	FCT = 0.214
Among populations within groups	1	1,382.899	71.951	34.51	FSC = 0.439^a^
Within populations	67	6,149.350	91.781	44.02	FST = 0.215^a^
Total	70	12,221.691	208.503		

^a^ *p* < 0.001.

**FIGURE 4:**
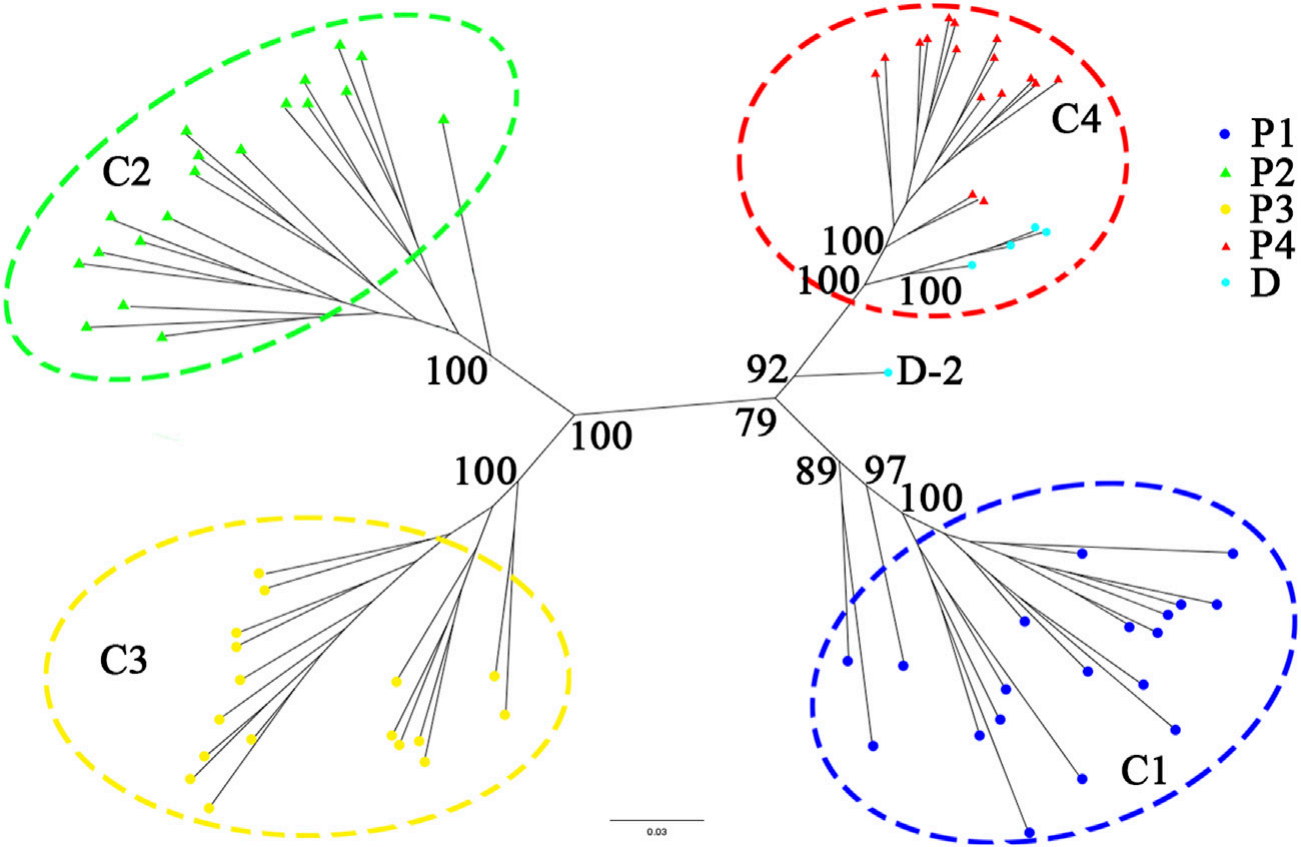
Phylogenetic tree constructed from nuclear RAD-seq data of the *C. granthamiana* and *C. albogigas.* At the nodes of the tree, maximum likelihood ultrafast bootstrap support values are shown for the four main clades.

A phylogenetic tree constructed from RAD-seq data (Figure 4) showed that individuals collected from the same population formed an independent monogroup, and four clades (C1–C4) could be found: the clade C1 (including all individuals of the population P1) was clustered together with C4 (including all individuals of the population P4), while C2 (including all the individuals of the population P2) was clustered together with C3 (including all the individuals of the population P3). Among the five cultivated samples at Sun Yat-sen University, D-1, D-3, D-4, and D-5 formed a monogroup and then clustered together with all the individuals sampled from P4, forming the clade C4 with a high support value (100%), while the last individual, D-2, was placed at the root of the clade C4 with a weak support value.

**FIGURE 5:**
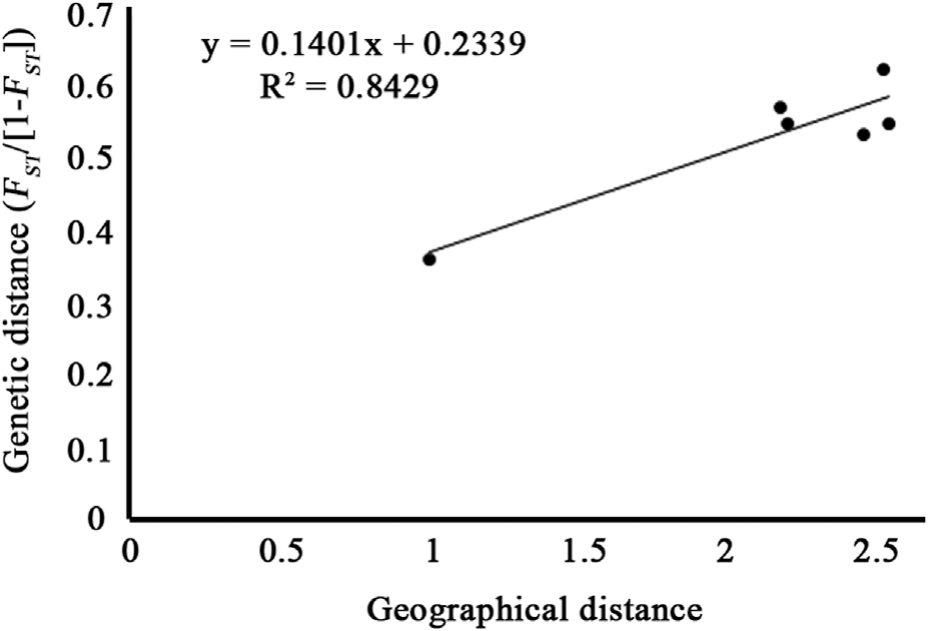
Mantel test between pairwise genetic distance (*F*_*ST*_/1-*F*_*ST*_) and geographical distance (ln) of the four populations of *C*. *granthamiana and C. albogigas.*

Pairwise *F*_*ST*_ estimation (Table 3) showed that the significant *F*_*ST*_ values were detected between each pair of populations, and the highest *F*_*ST*_ value was detected between the populations P2 and P4 (*F*_*ST*_ = 0.628), while the lowest *F*_*ST*_ was detected between P2 and P3 (*F*_*ST*_ = 0.365). The Mantel test showed that the genetic distance estimated using *F*_*ST*_/(1-*F*_*ST*_) between geographical distance (ln) was highly correlated (Figure 5, r = 0.912), although the *p*-value was not significant (*p* = 0.088).

**TABLE 3:** Pairwise *F*_*ST*_ (below the diagonal lines) and *N*_*M*_ (above the diagonal lines) among the four populations of *C*. *granthamiana*.

	P1	P2	P3	P4
P1	--	0.303	0.312	0.318
P2	0.575^a^	--	0.407	0.285
P3	0.552^a^	0.365^a^	--	0.312
P4	0.537^a^	0.628^a^	0.552^a^	--

^a^ *p* < 0.001.

### 3.4 Demographic history reconstruction

**FIGURE 6:**
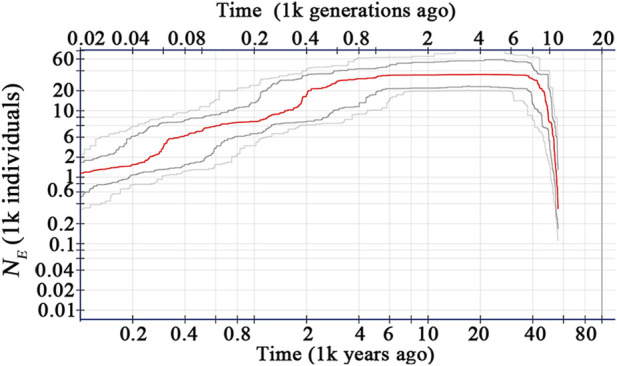
Estimates of the effective population size (*N*_*E*_) of *C*. *granthamiana*.

Based on the site frequency spectrum of the 7,152 variant sites, the effective population size (*N*_*E*_) of *C. granthamiana* was estimated to have expanded quickly since ca. 60,000 years ago (YA), reached a maximum level 40,000 YA, then remained stable till 4,000 YA, and kept declining to date (Figure 6).

## 4 Discussion

### 4.1 Treating *C. albogigas* as a synonym of *C. granthamiana*

The type specimen of *C. granthamiana* was recorded in Hong Kong, China, and we have not successfully collected fresh leaf tissue from Hong Kong yet. It is fortunate that in the Bamboo Garden of Sun Yat-sen University, both *C. albogigas* and *C. granthamiana* were cultivated and introduced from their respective localities. The plastid and nuclear tree supported that four of five cultivated individuals (D-1, D-3, D-4, and D-5) clustered together with *C. albogigas* (population P4) with high support values (Figures 2, 4), supporting that these four individuals should be transplanted from the type locality of *C. albogigas*. In the plastid tree, the last cultivated individual (D-2) clustered with P1-1, whose geographic location (Qiniangshan, Shenzhen, China) is just near Hong Kong, China, revealing that this individual should be introduced from the type locality of *C. granthamiana* (Hong Kong, China). It is interesting that D-2 is located at the root of clade P4 with a weak support value in the nuclear tree (Figure 4), while it is shown to be the admixture of the three groups in the ADMIXTURE analysis (Figure 1) and at the center of the other three clusters in the PCA (Figure 3). These data indicated an ancient position of D-2, which was introduced from Hong Kong, China.

In the *Flora of China* (Min and Bartholomew, 2007), *C. albogigas* was treated as a synonym of *C. granthamiana* despite some minor morphological differentiations between them (Chang, 1998). In this study, the only population of *C. albogigas* (P4) clustered together with the population P1 of *C. granthamiana* (Figures 1, 3), supporting this research. Although the plastid tree (Figure 2) showed that P4-1 was in subclade H2, P1-1, P2-1, and P3-3 were placed in subclade H3. The plastid genome of P4-1 could possibly result from plastid capture, which is frequently observed in many plant species (Liu, et al., 2020; Yang, et al., 2021). The occurrence of plastid capture, usually caused by repeated hybridization events, could also lead to the minor differences in morphological characters between *C. albogigas* and *C. granthamiana*.

### 4.2 Population differentiation within *C. granthamiana*

PCA and ADMIXTURE analysis (Figures 1, 3) showed that the four populations could be divided into three clusters (groups); AMOVA revealed that 21.47% genetic variation was maintained among groups (Table 2), and significant *F*_*ST*_ values were detected between each population pair (Table 3). These data supported a strong population structure within the species, possibly due to isolation by distance, as a high correlation was found between genetic and geographical distances (r = 0.912), and the lack of statistical significance (*p* = 0.088) could be caused by the limited number of populations (Figure 5). The demographic history construction (Figure 6) showed that the effective population size expanded since 60,000 YA, remained stable, and declined since 4,000 YA, suggesting that the species could be thriving during the Last Glacial Maximum and maintained its populations till 4,000 YA. At this time, human activities became more prevalent, which could have significantly affected the survival of the species and led to habitat fragmentation of many forest species (Aguilar, et al., 2006; Salmona, et al., 2017; Valenzuela-Aguayo, et al., 2020). The inbreeding coefficient *F*_*IS*_ of the species is estimated to be 0.065–0.174 (Table 1), suggesting a self-crossing tendency of the species that may have also resulted from habitat fragmentation. This self-crossing tendency could further facilitate the effect of genetic drift on small populations and lead to a strong population structure. In short, the species could have been widely distributed around Guangdong Province but experienced substantial retreat due to human disturbance over the past 4,000 years. It was only sporadically recorded in few locations up to date and then diverged significantly due to isolation by distance (Schaal and Leverich, 1996; Young, et al., 1996; Honnay, et al., 2005).

### 4.3 Genetic diversity and conservation of the species

It is inspiring that the four populations of *C. granthamiana* maintained a medium level of genetic diversity (*H*_*E*_: 0.120–0.162, Table 1), which is much higher than other endemic species, such as *Firmiana danxiaensis* (average *H*_*E*_: 0.115, RAD-seq, Chen, et al., 2023), *Primulina danxiaensis* (average *H*_*E*_: 0.064, RAD-seq, Chen, et al., 2021), and *Thuja sutchuenensis* (average *H*_*E*_: 0.082, RAD-seq, Yao et al., 2021), and lower than *Hemiculter leucisculus* in the Xinjiang Tarim River (average *H*_*E*_: 0.198, RAD-seq, Sun et al., 2022). Although only four populations were recorded, the largest geographical distance between them is close to 350 km; it is anticipated that more individuals (populations) will be found in the future. The significant differentiation between these populations and the strong population structure (Table 2; Figure 3) suggested that all the populations possess their own unique genetic variations and that more attention should be paid for their conservation.

## Data Availability

RAD raw data was deposited to the NCBI SRA database with BioProject PRJNA990452. Plastid genomes for nine *C. granthamiana* samples were deposited in GenBank with accession number OR224267-OR224275.
